# Supplementary material for: Reporter gene assays and chromatin-level assays define substantially non-overlapping sets of enhancer sequences
Source: BMC Genomics. 2023 Jan 13;24:17. doi: 10.1186/s12864-023-09123-9 (PMC9837977; doi:10.1186/s12864-023-09123-9)
Supplement: Supplementary file 3 — Additional file 3. [file 12864_2023_9123_MOESM3_ESM.pdf]

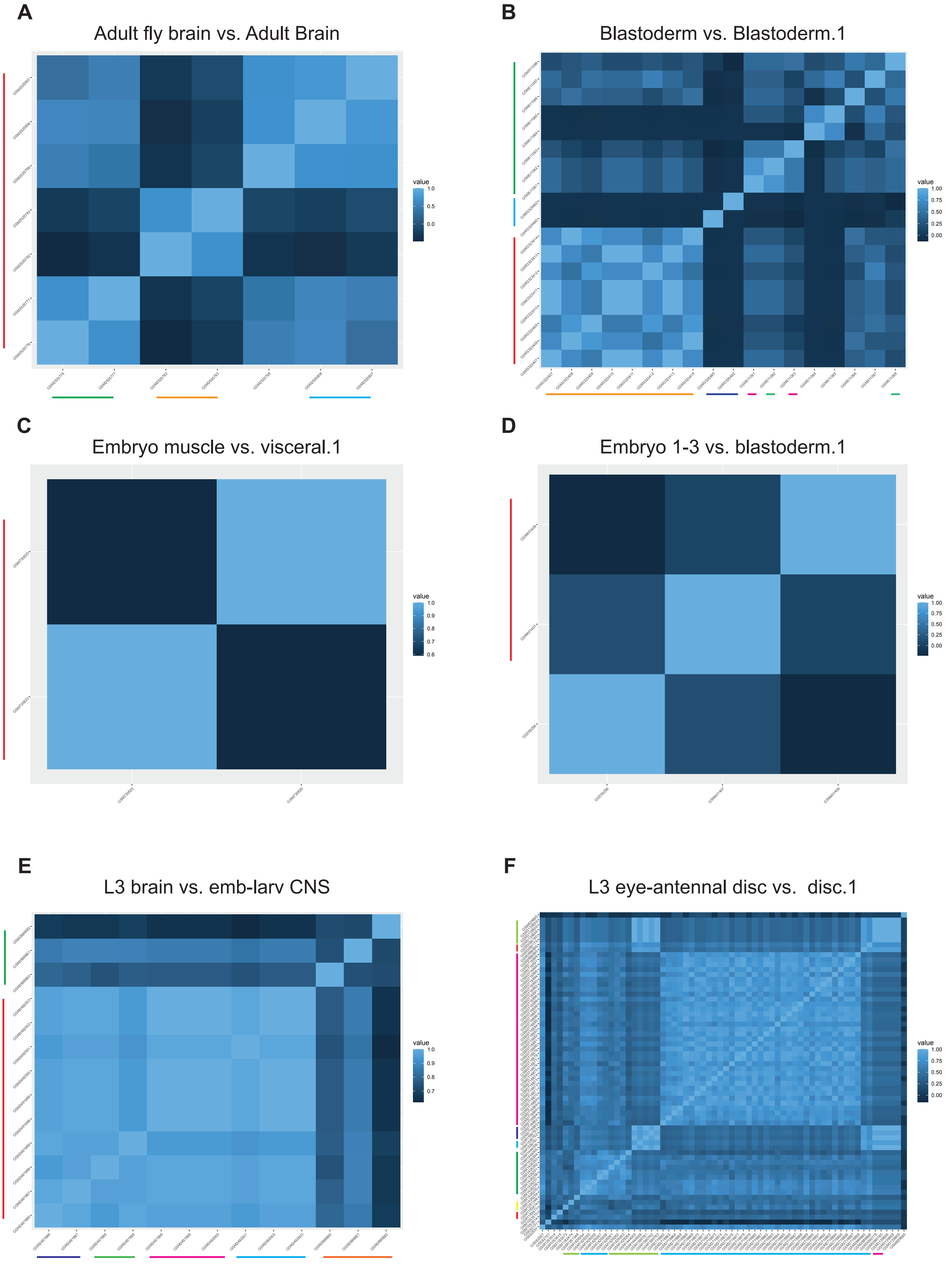
A
Adult fly brain vs. Adult Brain
value
1.0
0.5
0.0
B
Blastoderm vs. Blastoderm.1
value
1.00
0.75
0.50
0.25
0.00
C
Embryo muscle vs. visceral.1
value
1.0
0.9
0.8
0.7
0.6
D
Embryo 1-3 vs. blastoderm.1
value
1.00
0.75
0.50
0.25
0.00
E
L3 brain vs. emb-larv CNS
value
1.0
0.9
0.8
0.7
F
L3 eye-antennal disc vs. disc.1
value
1.00
0.75
0.50
0.25
0.00

Figure S1, Lindhorst and Halfon 2022

G

L3 eye disc vs. disc.1

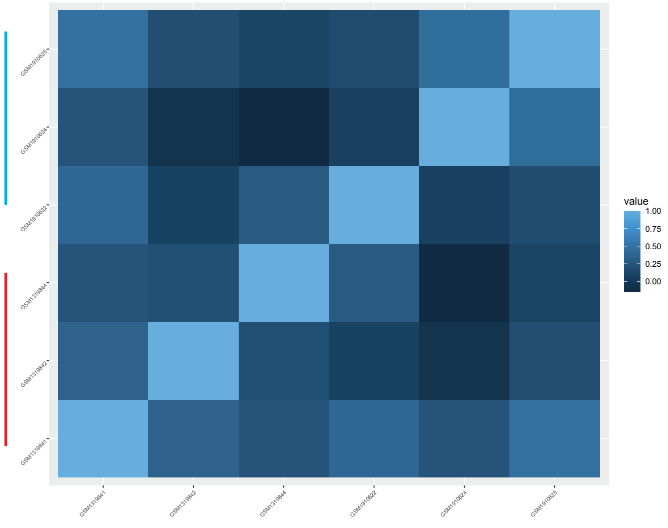

H

neuron vs. adult nervous system

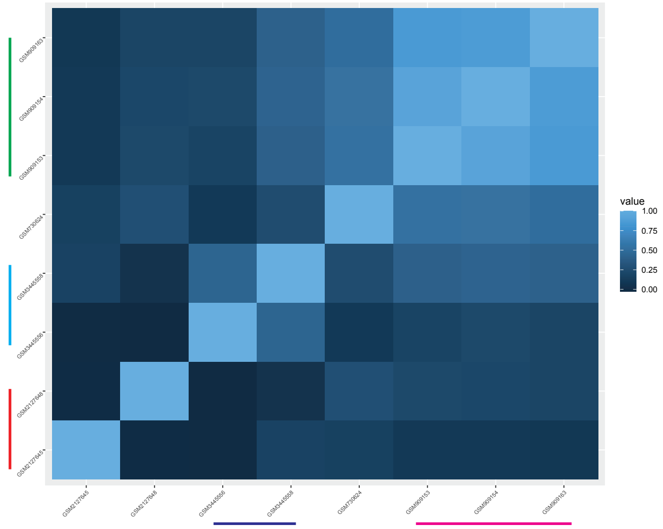

I

OSC vs. gonad

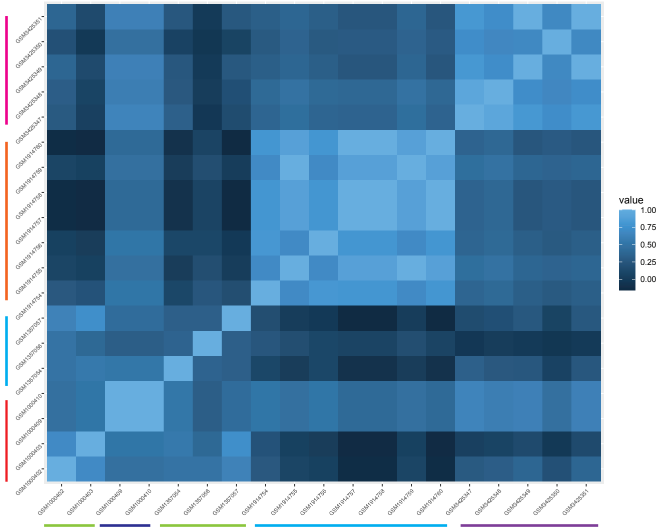

J

ovary vs. reproductive.1

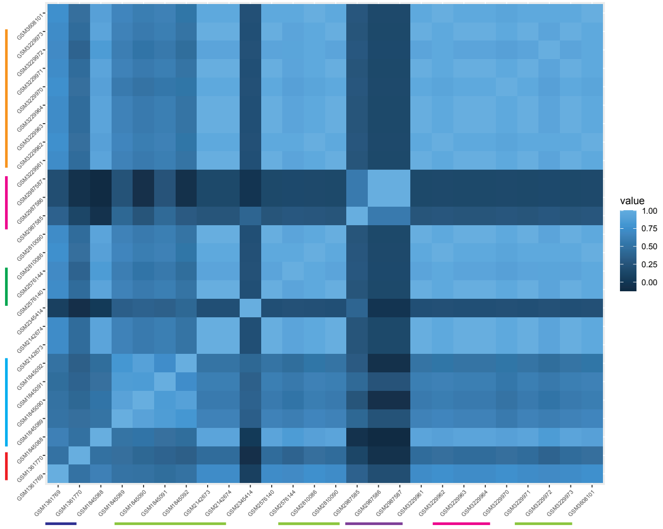

Figure S1: Correlations between REDfly enhancers overlapping enhancers from EnhancerAtlas subsets.

Correlations between EnhancerAtlas subsets and REDfly enhancers from (A) Adult fly brain/Adult brain; (B) Blastoderm/Blastoderm.mapping1; (C) Embryo muscle/visceral.mapping1; (D) Embryo 1-3/Blastoderm.mapping1; (E) L3 brain/embryo-larval CNS; (F) L3 eye-antennal disc/disc.mapping1; (G) L3 eye disc/disc.mapping1; (H) neuron/adult nervous system; (I) OSC/gonad; (J) ovary/reproductive.mapping1. Correlations are scaled from strong (light blue) to weak (dark blue). Common experiment series as provided in GEO are on the y-axis, with colored bars indicating common sets. Assay types are on the x-axis with colored bars indicating similar assay types. For details, see Table S1d. On the whole, experiment series (y-axis) is a stronger driver of correlation than assay method (x-axis).
